# M2 microglia promotes neurogenesis and oligodendrogenesis from neural stem/progenitor cells via the PPARγ signaling pathway

**DOI:** 10.18632/oncotarget.15774

**Published:** 2017-02-28

**Authors:** Jichao Yuan, Hongfei Ge, Wei Liu, Haitao Zhu, Yaxing Chen, Xuan Zhang, Yang Yang, Yi Yin, Weixiang Chen, Wanjiang Wu, Yunfeng Yang, Jiangkai Lin

**Affiliations:** ^1^ Department of Neurosurgery and Key Laboratory of Neurotrauma, Southwest Hospital, Third Military Medical University, Chongqing 400038, China; ^2^ Department of Neurology, Southwest Hospital, Third Military Medical University, Chongqing 400038, China

**Keywords:** NSPCs, differentiation, M2 microglia, 15d-PGJ2, PPARγ

## Abstract

Neural stem/progenitor cells (NSPCs) are an important source of cells for cell replacement therapy after nerve injury. How to induce NSPCs differentiation towards neurons and oligodendrocytes is a challenging issue in neuroscience research. In the present study, we polarized microglia into M1 and M2 phenotype, used their supernatants to induce NSPCs differentiation, and investigated the effects of different microglia phenotypes on NSPCs differentiation and their mechanisms. We discovered that, after exposure to M1 phenotype supernatant, NSPCs differentiated into fewer Tuj-1+ and Olig2+ cells, but more GFAP+ cells. Meanwhile, a significantly increased number of Tuj-1+ and Olig2+ cells and smaller number of GFAP+ cells were generated by M2 microglia supernatant-induced NSPCs differentiation. We also observed that 15d-PGJ_2_, an endogenous ligand of PPARγ, was elevated in M2 phenotype supernatant and could activate PPARγ expression in NSPCs, whereas use of the PPARγ inhibitor GW9662, could reduce the percentage of differentiated neurons and oligodendrocytes. Our study results confirm that M2 microglia supernatant can activate the PPARγ signaling pathway and promote neurogenesis and oligodendrogenesis from NSPCs differentiation. The present study provides a further theoretical basis for induction of NSPCs oriented differentiation.

## INTRODUCTION

Neural stem/progenitor cells (NSPCs) have a strong capacity for self-proliferation and differentiation. Thus they have great potential for use as replacement cells and play an important role in neural repair after central nervous system (CNS) injury [1]. However, due to the loss of various nutritional factors and the persistent secondary inflammation, more NSPCs are differentiated into astrocytes and ultimately form glial scars, which inhibit axon regeneration [2–4]. Therefore, it is of great importance to find an effective way to promote neurogenesis and oligodendrogenesis from NSPCs differentiation.

Inflammation is a key factor affecting NSPCs differentiation [5]. As resident immune cell in the CNS, microglia can be rapidly activated after injury, and participate in local inflammatory reaction [6]. Microglia can exert beneficial [7] or harmful [8, 9] effects, and this mainly depends on the two different microglia subtypes. Proinflammatory M1 microglia activated by tumor necrosis factor alpha (TNF-a) and interferon γ (IFN-γ) via classical pathways disrupts the internal environment. In contrast, anti-inflammatory M2 microglia activated by interleukin 4 (IL-4) and interleukin 13 (IL-13) via selective pathways plays a neuroprotective role [10, 11]. It has been reported that microglia activated by IL-4 can promote neurogenesis and oligodendrogenesis from NSPCs differentiation [12], but the specific mechanism of action is unclear.

Prostaglandin D_2_ (PGD_2_), a type of cyclopentenone prostaglandin, is formed by the cyclooxygenation of arachidonic acid. 15-Deoxy-Δ12,14-prostaglandin J_2_ (15d-PGJ_2_) is the ultimate metabolite of PGD_2_ through spontaneous nonenzymatic dehydration followed by isomerization and is an endogenous ligand for PPARγ [13]. 15D-PGJ_2_ possesses anti-inflammatory properties and plays a protective role in CNS injury [14–16]. It has been reported that anti-inflammatory macrophages could release 15d-PGJ_2_ [17–19] and that 15d-PGJ_2_ could promote the proliferation of NSPCs via the PPARγ pathway [20]. Therefore, we speculated that 15d-PGJ_2_ and PPARγ participate in M2 microglia-induced NSPCs differentiation.

In the present study, M1 and M2 microglia supernatants are used to induce NSPCs differentiation; and the differentiation into neurons, oligodendrocytes, and astrocytes is observed; the possible underlying mechanisms are also explored. This study provides an experimental evidence for improving the internal environment of the body after nerve injury and promoting NSPCs differentiation, and it furnishes certain interventional measures and targets for neural function recovery at later stages after CNS injury.

## RESULTS

### Polarization of microglia

**Figure 1:**
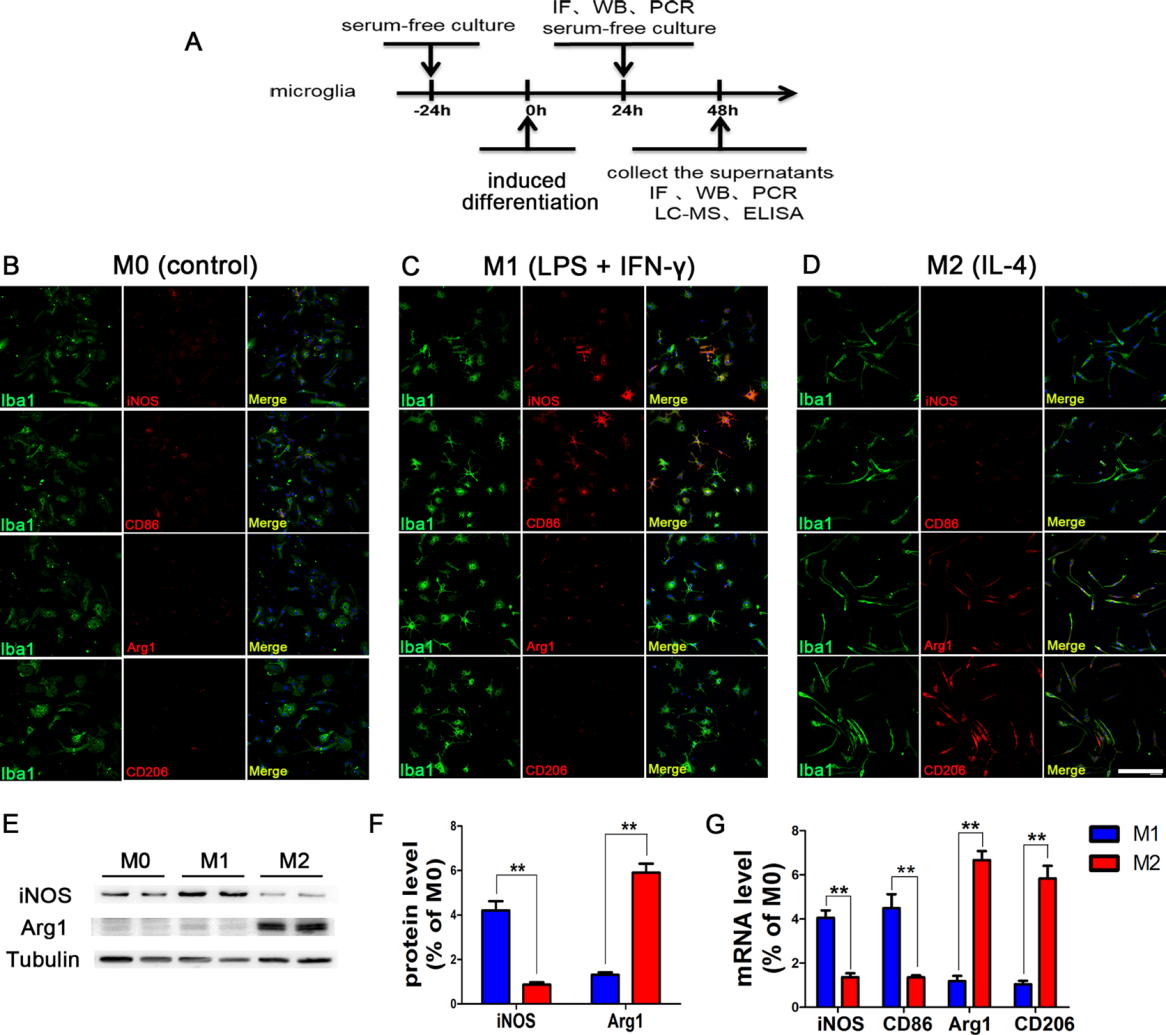
Phenotypic changes of polarized microglia under different intervention conditions (**A**) The scheme of the experimental timeline for microglia. Phenotypic changes of polarized microglia induced by serum-free culture medium (**B**), LPS+IFN-γ (**C**), and IL-4 (**D**) for 24 h. M1 subtype-specific markers iNOS and CD86 are more highly expressed in LPS+IFN-γ polarized microglia, while M2 subtype-specific markers CD206 and Arg1 are more highly expressed in IL-4 polarized microglia. The results of WB (**E**, **F**) and PCR (**G**) assays were consistent. *N* = 8, bar = 50 μm, **P < 0.01.

The M0 microglia induced by serum-free culture medium were of irregular morphology; the expression levels of M1 subtype markers (iNOS and CD86) and M2 subtype markers (CD206 and Arg1) were all low, as the microglia had not yet been activated. After induction by LPS+IFN-γ for 24 hours, the microglia became round and the number of microglia expressing iNOS and CD86 was significantly increased, whereas the expression levels of both CD206 and Arg1 were low. After induction by IL-4, the microglia exhibited long and spindle-like morphology, and the number of cells expressing CD206 and Arg1 was significantly increased, while the expression levels of iNOS and CD86 were relatively low (Figure 1). WB and PCR assays were consistent with these findings. These results demonstrated that we had successfully polarized microglia cultured *in vitro* into M1 and M2 subtypes.

**Figure 2:**
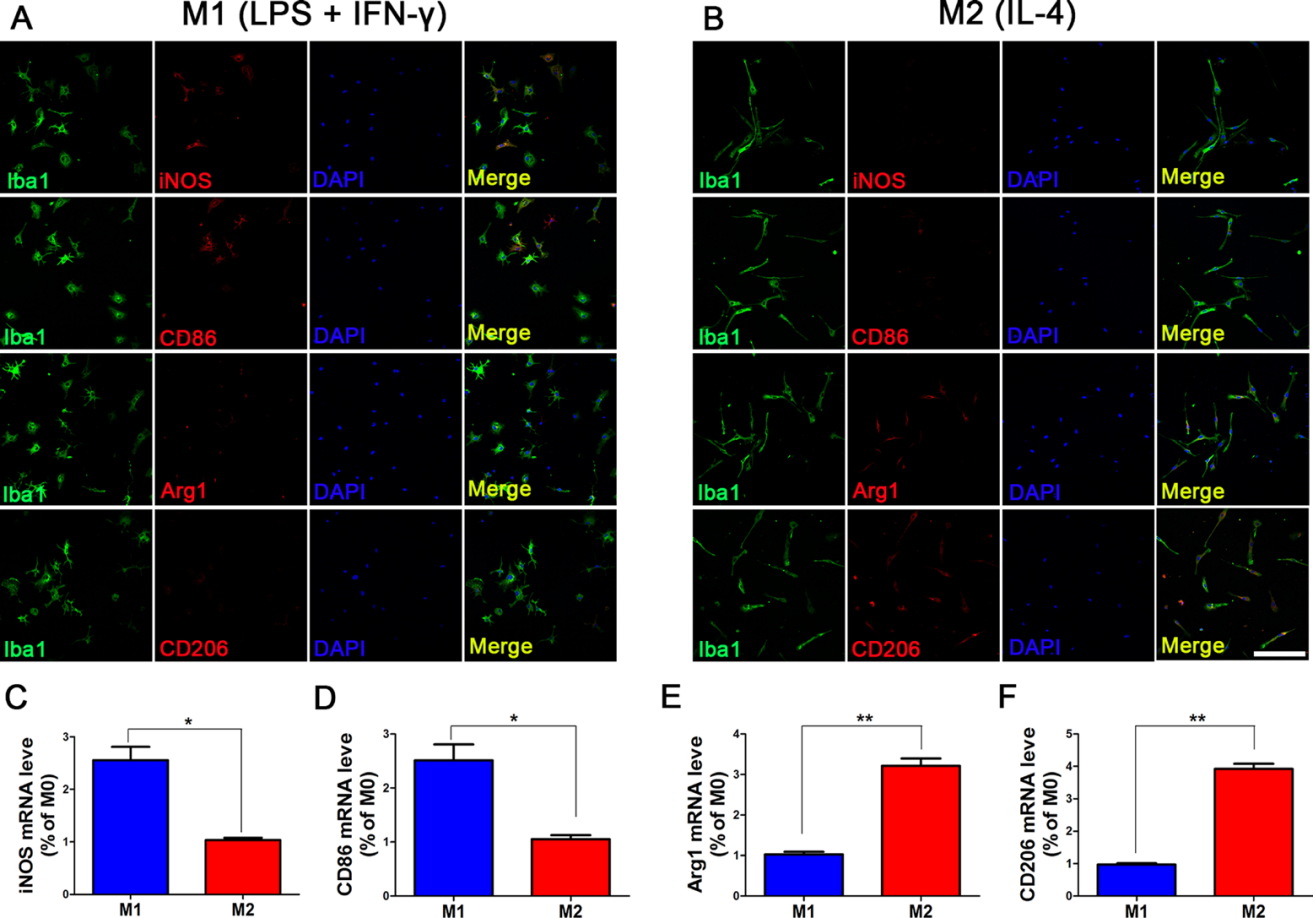
Phenotype maintenance of M1 and M2 microglia at 24 h after removal of intervention After microglia were polarized by LPS+IFN-γ and IL-4 for 24 h, LPS+IFN-γ and IL-4 were replaced with serum-free culture medium, and microglia were cultured for another 24 h. Approximately 50% of microglia polarized by LPS+IFN-γ still expressed M1 subtype-specific markers iNOS and CD86, and iNOS mRNA and CD86 mRNA were still highly expressed (**A**). Approximately, about half of the microglia polarized by IL-4 expressed the M2 subtype-specific markers CD206 and Arg1 (**B**). The expression levels of these two markers differed from those in M0 microglia, and the differences were statistically significant (**C–F**). *N* = 8, bar = 50 μm. *P < 0.05, **P < 0.01.

To prove that microglia can maintain M1 and M2 phenotype without intervention, we measured the expression levels of M1 and M2 subtype markers after the intervening factors were removed (Figure 2). An immunofluorescence assay revealed that the levels of iNOS and CD86 expression in LPS+IFN-γ induced cells were somewhat reduced compared to those before the intervention was removed, but approximately half of the cells continued to express M1 subtype markers. Additionally, approximately 50% of M2 microglia induced by IL-4 still expressed CD206 and Arg1, and the levels of mRNA were also elevated. These data indicated that the supernatants we collected contained high levels of M1 and M2 microglia secretions.

### NSPCs characteristics

**Figure 3:**
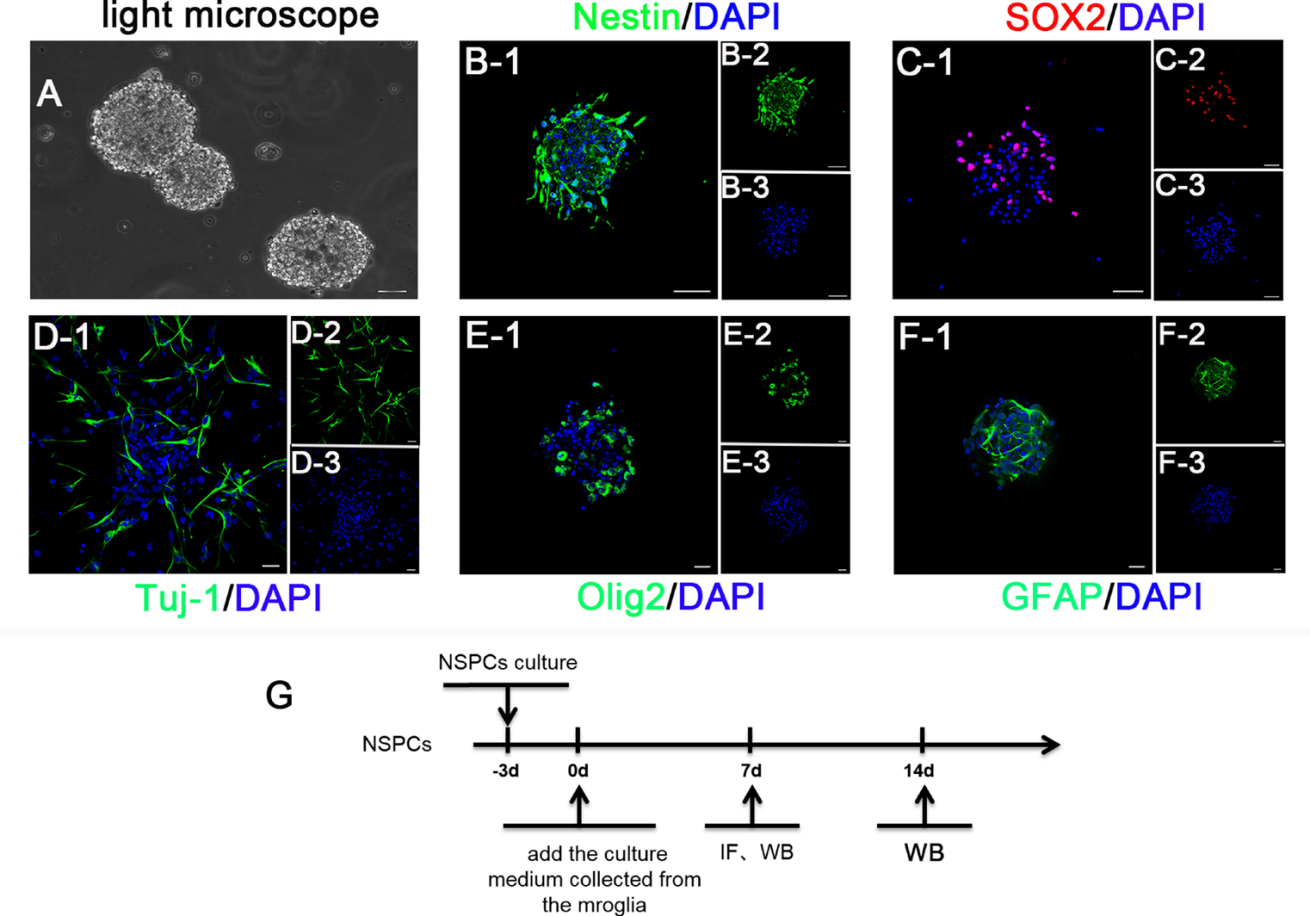
(**A**) The phase contrast photo showed the suspended growth of neurospheres. (**B**) and (**C**) The immunostaining depicted isolated cells expressing Nestin (green) and SOX2 (red). (**D**–**F**) The immunofluorescence identification for the differentiation potential of NSPCs into neurons (Tuj-1), astrocytes (GFAP), and oligodendrocytes (Olig2), respectively. Cell nuclei was stained with DAPI in blue. (**G**) A scheme of the experimental timeline for NSPCs. Bar = 20 μm.

After three days of culture, NSPCs were globular in shape and grew in a suspended manner. Approximately 60% of cells expressed nestin and SOX2, which are the NSPCs-specific markers. After adherence to walls and differentiation for 14 days, the neurospheres expressed neuronal marker Tuj-1, oligodendrocyte marker Olig2, and astrocyte marker GFAP (Figure 3). The terminal differentiation markers (MAP2 and O4) were also consistent with these findings (Supplementary Figure 1). These results showed that the cells we cultured had improved differentiation potential and could be differentiated into various major cell subtypes in the central nervous system.

### M0, M1 and M2 microglia supernatants do not affect the apoptosis and proliferation of NSPCs

With apoptosis assay and LDH release assay, we found that there were no statistically significant differences among M0, M1 and M2 microglia on day 3, 7 and 14 (Supplementary Figure 2). These data indicated that M0, M1 and M2 microglia had no different influence on the death/survival of NSPCs. Similarly, CCK-8 proliferation assay (Supplementary Figure 2) showed that different phenotypes had no different influence on the proliferation of NSPCs.

### M2 microglia supernatant promotes NSPCs differentiation towards neurons and oligodendrocytes

**Figure 4:**
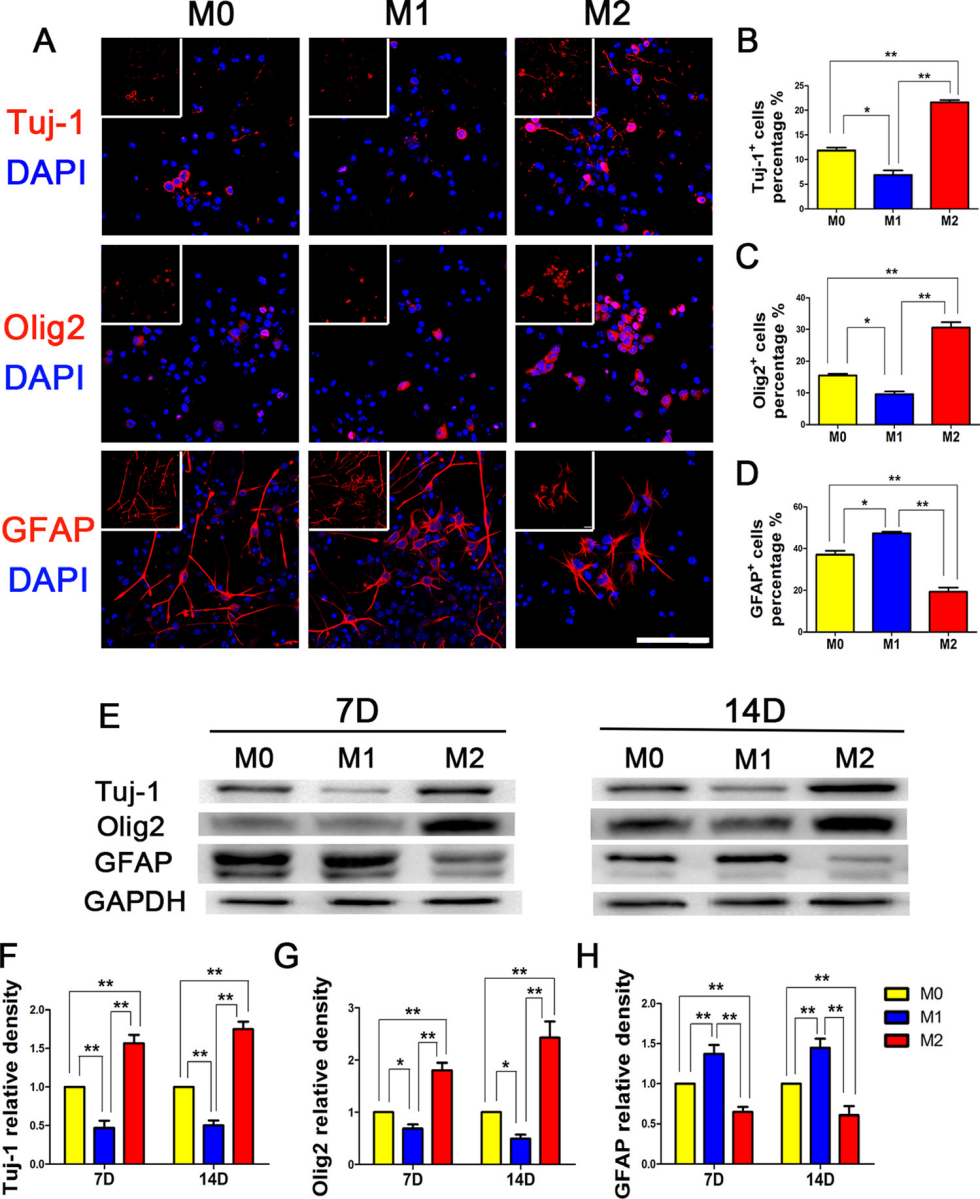
NSPCs differentiation induced by different microglia supernatants M0, M1, and M2 microglia supernatants were separately collected and added to 1% FBS to induce NSPCs differentiation for 14 days. (**A**) Immunofluorescence, (**B**–**D**) Statistical analysis for the differentiation percentage of neurons (Tuj-1), oligodendrocytes (Olig2) and astrocytes (GFAP) from NSPCs, respectively. WB results (**E**) showed that M2 microglia supernatant up-regulated Tuj-1 and Olig2 expression levels and reduced GFAP expression in NSPCs. The differences in Tuj-1, Olig2 and GFAP+ cells induced by M0, M1, and M2 microglia supernatants were all statistically significant (**F–H**). *N* = 8, bar = 50 μm. *P < 0.05, **P < 0.01.

Three days after NSPCs extraction, we added supernatants from polarized microglia to induce NSPCs adherence and differentiation (Figure 4). We found that on day 14 of differentiation, approximately 40% of NSPCs induced by M0 supernatant in the control group were differentiated into GFAP+ astrocytes, with Tuj-1+ and Olig2+ cells each accounting for approximately 10%. After induction by M1 supernatant, the percentages of Tuj-1+ and Olig2+ cells were further reduced, and more GFAP+ differentiated cells were generated. In contrast, NSPCs induced by M2 supernatant were more likely to differentiate into Tuj-1+ cells (approximately 20%), Olig2+ cells (approximately 30%), and significantly decreased GFAP+ cells (only approximately 20%). A WB assay showed that the expression levels of Tuj-1 and Olig2 were significantly elevated at day 7 and 14 after M2 microglia induction compared to M1 microglia supernatant induction, while the level of GFAP expression was significantly reduced. These results suggested that M2 microglia supernatant increased the numbers of neurons and oligodendrocytes generated by NSPCs differentiation, while reducing the differentiation ratio of astrocytes.

### Higher level of 15d-PGJ2 in M2 microglia supernatant

**Figure 5:**
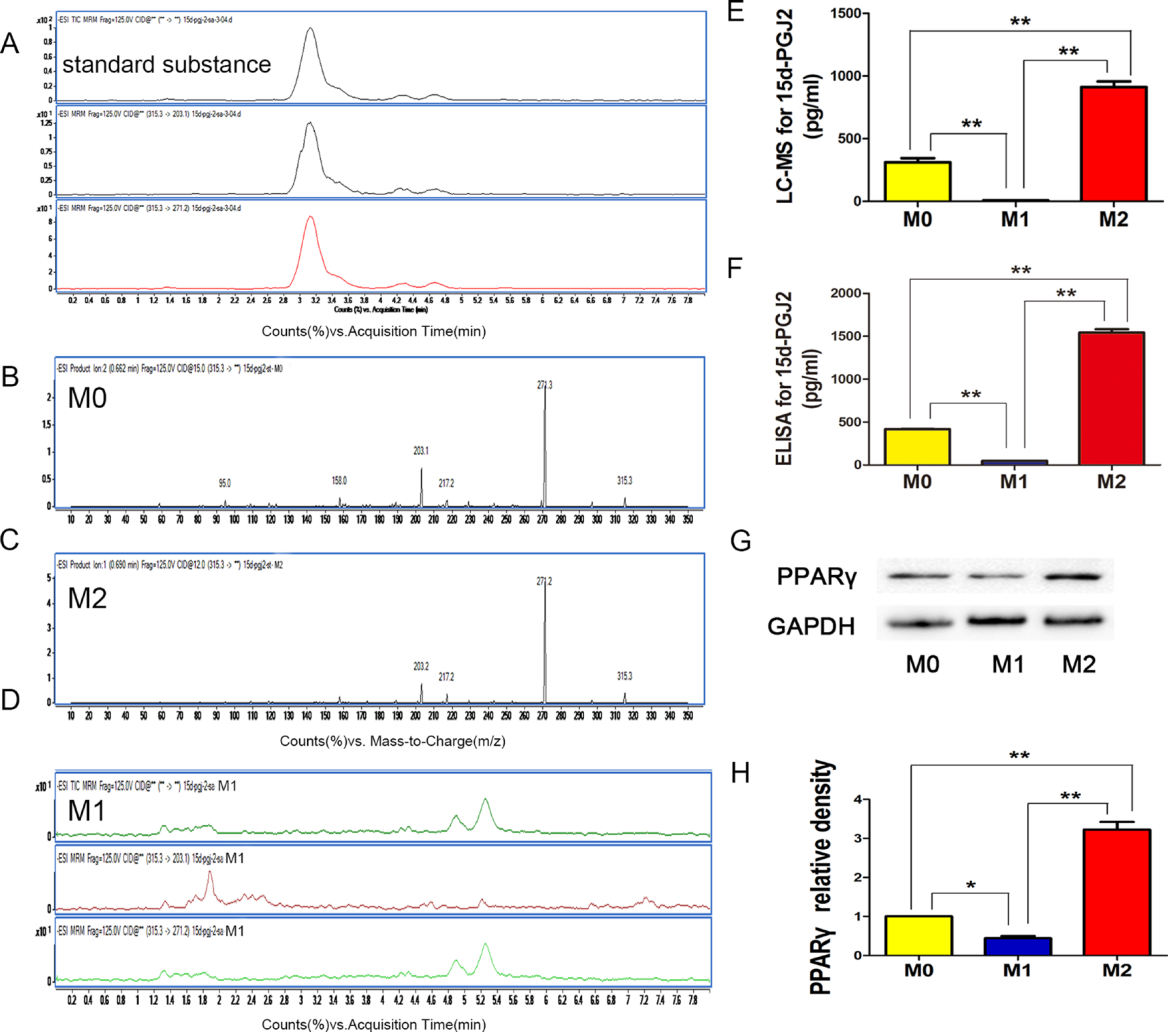
15D-PGJ2 released in M0, M1, and M2 microglia (**A**) With 12 V or 15 V voltage, we divided the solvent system (315.3 molecular weight, M-H-) into the quantitative ion (271.2 molecular weight) and the qualitative ion (203.1 molecular weight), respectively. (**B**–**D**) LC-MS analysis for 15d-PGJ_2_ in M0, M1, and M2 microglia. (**E**) Statistical analysis for level of 15d-PGJ_2_ with LC-MS. (**F**) Statistical analysis for 15d-PGJ_2_ level with ELISA. (**G**, **H**) WB for PPARγ expression in NSPCs induced with different microglia supernatants. *N* = 8, *P < 0.05, **P < 0.01.

We further explored the amount of 15d-PGJ_2_ during microglia polarization, this compound has been identified as a natural ligand for PPARγ (Peroxisome proliferator-activated receptor γ). LC-MS (Figure 5A–5E) and ELISA (Figure 5F) analyses detected ever little 15d-PGJ_2_ in the M1 supernatant, whereas the amount of 15d-PGJ_2_ in the M2 supernatant was very abundant (Figure 5E, 5F).

### PPARγ participates in NSPCs differentiation induced by M2 microglia supernatant

To investigate the mechanism by which 15d-PGJ_2_ from M2 microglia affects NSPCs differentiation, we studied PPARγ expression in NSPCs on day 14 (Figure 5G, 5H). We found that the expression of PPARγ in M2 supernatant-induced NSPCs was increased, whereas its expression in M0 and M1 supernatant-induced NSPCs was was low. These results were consistent with 15d-PGJ_2_ released. 15D-PGJ_2_ is an endogenous ligand of PPARγ. Therefore, we speculated that 15d-PGJ_2_ activated PPARγ to promote NSPCs differentiation into neurons and oligodendrocytes.

### Inhibition of PPARγ reduces the percentage of neurons and oligodendrocytes generated by NSPCs differentiation

**Figure 6:**
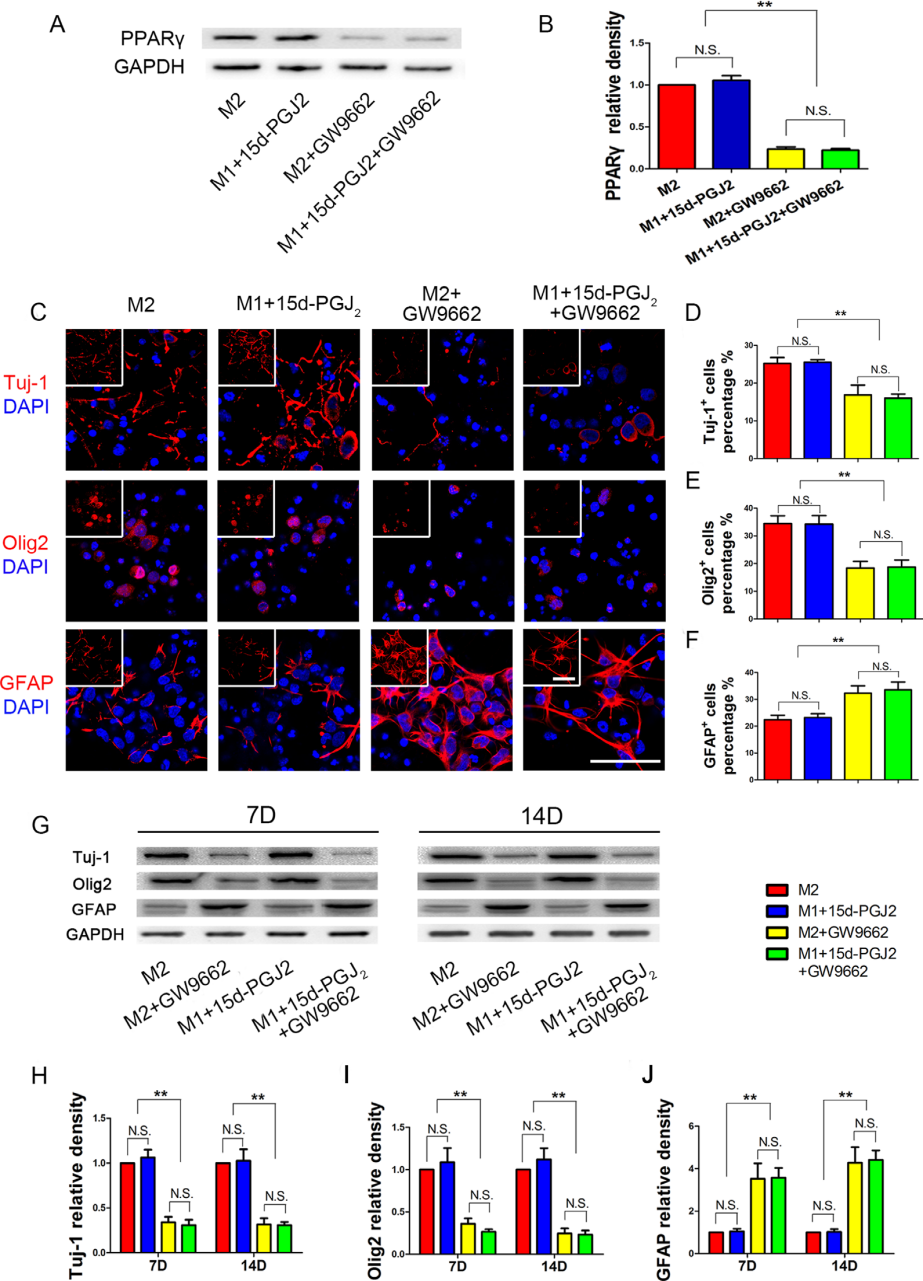
Inhibition of PPARγ reduced NSPCs differentiation induced by M2 microglia towards neurons and oligodendrocytes GW9662 (30 μmol/ml) was added 1h in advance to block PPARγ activity, and then M2 supernatant or M1 supernatant + 15d-PGJ_2_ (1 ngl/ml, the same concentration with M1 supernatant) were added to induce NSPCs differentiation. During the entire differentiation process, the concentrations of 15d-PGJ_2_ and GW9662 were maintained at 1 ngl/ml and 30 μmol/ml, respectively. (**A**, **B**) WB showed PPARγ expression on day 14 during NSPCs differentiation. (**C**) Immunofluorescence, (**D**–**F**) Statistical analysis for neurons (Tuj-1), oligodendrocytes (Olig2) and astrocytes (GFAP) differentiation percentage from NSPCs, respectively. (**G**) WB results. (**H**–**J**) Statistical analysis of the WB. *N* = 8, bar = 50 μm. N.S. indicates no significant difference, *P < 0.05, **P < 0.01.

To verify this hypothesis, we used a 15d-PGJ_2_ monomer and the PPARγ inhibitor GW9662 to intervene in NSPCs differentiation (Figure 6). We found that 15d-PGJ_2_ activated PPARγ expression and GW9662 suppressed PPARγ expression. There were no significant differences in Tuj-1, Olig2 or GFAP protein expression between the M2 group and the M1+15d-PGJ_2_ group. These data indicated that 15d-PGJ_2_ could promote neurogenesis and oligodendrogenesis. With GW9662 inhibited, the percentage of GFAP+ cells significantly increased. These results demonstrated that inhibition of PPARγ could promote NSPCs differentiation towards astrocytes, and limit the differentiation towards neurons and oligodendrocytes.

## DISCUSSION

Our study discovers that, when induced by anti-inflammatory M2 microglia supernatant, NSPCs differentiated significantly more into neurons and oligodendrocytes and less into astrocytes. M2 supernatant promotes NSPCs differentiation through the secretion of 15d-PGJ_2_, which can activate PPARγ in NSPCs nucle. Our research confirm that M2 microglia can promote neurogenesis and oligodendrogenesis from NSPCs differentiation through the PPARγ pathway.

As the resident immune cells in CNS, microglia are key mediators of secondary inflammation. M2 phenotypic microglia can improve neural repair after CNS injury [21–25]. In the present study, microglia polarized by IL-4 became long and spindle-like, the expression levels of M2 subtype markers significantly increased, and the percentage of oligodendrocytes and neurons differentiated from NSPCs exposed to M2 microglia supernatant were increased. This result is consistent with a previous report [12]. At the same time, we also found that M1 microglia induced by LPS+IFN-γ promoted astrocyte differentiation, while Butovsky discovered that IFN-γ activated microglia showed a bias towards neurogenesis. We found that the discrepancy was caused by different cell models. Butovsky et al. used microglia activated by IFN-γ and used cover slips coated with matrigel to co-culture microglia and NSPCs [12]. However, our model combined LPS+IFN-γ to promote microglia activation; we then collected the supernatant to prepare the culture medium used to induce differentiation. When inoculating NSPCs, we avoided using dishes pretreated with any biomaterial substrates; because dishes pretreated with poly-L-ornithine could induce NSPCs differentiation towards neurons and oligodendrocytes [26]. Zhang et al. used a similar model and reached a relatively consistent conclusion [27]. The research indicated that M2 microglia could assist NSPCs migration to the injury region and promote neurogenesis.

There remains a lack of in-depth studies on how M2 microglia promotes neurogenesis and oligodendrogenesis. In this study, we found that the released 15d-PGJ_2_ in the M2 microglia supernatant was significantly increased compared to that in the M1 supernatant. Additionally, 15d-PGJ_2_-rich supernatant could activate PPARγ in NSPCs, significantly increase the number of Tuj-1+ and Olig2+ cells generated during NSPCs differentiation, and reduce the number of GFAP+ cells. However, after intervention with the PPARγ inhibitor GW9662, NSPCs differentiation exhibited the opposite phenomenon. These data suggest that 15d-PGJ_2_ and PPARγ are important factors for M2 microglia promotion of NSPCs differentiation.

15D-PGJ_2_ is derived from PGD_2_, a major cyclooxygenase 2 product synthesized in a variety of tissues under inflammatory conditions [28]. As the receptor for 15d-PGJ_2_, PPARγ is a key factor in glucose homeostasis, lipid metabolism and the regulation of cellular functions in a ligand-dependent manner [29–31]. PPARγ also plays an important role in the process of NSPCs differentiation. Morales et al. significantly increased the numbers of MAP-2- and GFAP-positive cells by using the PPARγ agonist pioglitazone, and the addition of GW9662 completely abolished these effects [32]. It has also been reported that PPARγ agonists can increase the quantities of TuJ1- and CNPase-positive cells, while the PPARγ-selective antagonist GW9662 can reduce quantities of TuJ1- and CNPase-positive cells [33, 34]. Further, it has been reported that PPARγ can promote NSPCs differentiation towards astrocytes [35]. The results of our experiments indicate that M2 microglia can activate PPARγ expression in NSPCs and promote neurogenesis. Additionally, these data demonstrate that PPARγ plays an important role in NSPCs differentiation.

In summary, the present study illustrates that M2 microglia supernatant can increase the percentage of neurons and oligodendrocytes generated by NSPCs differentiation, and decrease the number of differentiated astrocytes. M2 microglia promotes NSPCs differentiation by activating the PPARγ signaling pathway. Our experiment provides further experimental support and alternative options for promoting NSPCs-oriented differentiation.

## MATERIALS AND METHODS

### Animal

This study was performed in accordance with the China's animal welfare legislation for the care and use of animals and approved by the Third Military Medical University in Chongqing, China. We did our best to minimize the number of animals and decrease their suffering. E14.5 ICR mice were sacrificed after anesthetized with pentobarbital (60 mg/kg intraperitoneally).

### Microglia culture

The cortex of newborn mice was dissected and the cerebral pia mater was stripped under an anatomical microscope. The tissue was digested in 0.125% trypsin for 8 min and stopped from digestion with 10% fetal bovine serum (FBS). Then, the digested material was filtered with 200-mesh metal strainer. After centrifugation of the filtrate, a cell suspension was prepared in DMEM/F12 containing 1% double antibodies and 10% FBS, placed into a 5% CO_2_ incubator and incubated at 37°C for 12 h; the suspension was replaced after the cells adhered to the walls. Thereafter, the culture medium was replaced once every three days. On day 14, the culture was placed on a shaker at 250 rpm for 1 h, the supernatant was collected, and microglia were obtained. As identified by Iba1 staining, the purity of the microglia preparation was greater than 95%.

### Polarization of microglia and preparation of NSPCs conditional culture media

Purified microglia were spread on a six-well plate at the concentration of 10^6^/well, serum deprived, and cultured for 24 h. Then, serum-free culture medium was used to induce M0 microglia, serum-free culture medium containing lipopolysaccharides (LPS, 100 ng/ml) + IFN-γ (25 μg/ml) was used to induce M1 microglia, and serum-free culture medium containing IL-4 (20 μg/ml) was used to induce M2 microglia. The microglia were cultured under the three culture medium conditions for 24 h, after which the media was replaced, and then serum-free culture media was added to continue culturing. After 24 h, 1% FBS was added to the collected the supernatants, and the culture media was used to induce NSPC differentiation.

### NSPCs culture

The mouse embryos were obtained from the 14.5-day pregnant mice. The cortex was dissected and cut into pieces, digested in 0.125% trypsin for 8 min, stopped from digestion with soybean trypsin inhibitor, and filtered with a 100 μm Nylon cell strainer. The cells were re-suspended in NSPC culture medium at an initial concentration of 1*10^5^ cells/mL, and cultured in a 5% CO_2_ incubator at 37°C. The NSPC culture medium was DMEM/F12 complete medium containing 20 ng/ml recombinant murine FGF (PeproTech, 450–33), 20 ng/ml recombinant murine EGF (PeproTech, 315–09), and 2% B-27 supplement (Life Technologies, 17504–044). After culture for three days, the culture was centrifuged at 800 rpm for 5 min, and then the cells were re-suspended in culture medium for induced NSPC differentiation. 15D-PGJ_2_ (Sigma, D8440, 1 ng/ml) and GW9662 (Sigma, M6191, 30 μmol/ml) were added to the conditional culture media. Half of the volume of culture medium for induced differentiation was replaced every three days.

### Apoptosis assay

Annexin-V staining was used to detect the death/survival of NSPCs with an Annexin V-FITC Apoptosis Detection Kit (Beyotime) and processed in accordance with the manufacturer recommendation. 2 * 10^6^ cells were cultured with NSPC culture medium for 3 days. Then supernatants collected from M0, M1, M2 microglia were added to induce NSPC differentiation. On day 3, 7 and 14, adherent cells were collected with StemPro^R^ Accutase^R^ Cell Dissociation Reagent (Life Technologies, A1110501). Cells were incubated with Annexin V-FITC at room temperature for 10 minutes and re-suspended with binding buffer. Then Propidium Iodide was added into suspension for 5 minutes on ice. Cells were analyzed using a FACScan flow cytometer.

### lactate dehydrogenase (LDH) release assays

Cell necrosis were measured with lactate dehydrogenase (LDH) release assays. 100 μl of cell suspension (~10000 cells/well) was dispensed in a 96-well cell culture cluster with NSPC culture medium for 3 days. Then NSPCs differentiation was induced with different phenotypes microglia supernatants. Supernatants were collected on day 3, 7 and 14. Then cells were lysed with 2% Triton X-100 for 15 min to release all LDH. The cell lysis were used as positive controls to determine the maximal LDH release. LDH release was detected using the absorbance of the culture medium at a test wavelength of 450 nm with a microplate reader according to the manufacturer's instructions.

### Cell proliferation assay

Cell counting Kit-8 was used to assess cell proliferation. 10^4^ cells/well NSPCs were cultured in a 96-well plate and induced differentiation with M0, M1, M2 supernatants. On day 3, 7 and 14, the cells were incubated with 10% (v/v) WST solution for 2.5 h at 37°C. Then, the absorbance of the culture medium at a test wavelength of 450 nm was determined using a microplate reader and a reference wavelength of 630 nm as well.

### Immunofluorescence

Intervened cells were fixed in 4% paraformaldehyde, penetrated with 0.1% Triton X-100, blocked with 5% bovine serum albumin (BSA) and incubated with primary antibodies at 4°C overnight. Afterwards, the cells were stained with fluorescein-labeled secondary antibodies and 4′,6-diamidino-2-phenylindole dihydrochloride (DAPI) under dark conditions, sealed on the slides, and observed under a laser confocal microscope. The respective concentrations of primary antibodies were: Iba1 (wako, Lot: 019–19741, 1:1000), CD86 (BD, Lot: 553689, 1:200), iNOS (BD, Lot: 610328, 1:200), CD206 (Santa, sc-34577, 1:100), Arg1 (Santa, sc-18351, 1:100), Nestin (Abcam, ab6142, 1:200), SOX2 (Abcam, ab97597, 1:200), Tuj-1 (Abcam, ab78078, 1:200), Olig2 (Abcam, ab109186, 1:100), GFAP (Abcam, ab7260, 1:1000), MAP2 (Abcam, ab5392, 1:500), O4 (Sigma, O7139, 1:100), A minimum of four images was captured using a 20X objective on a Zeiss microscope (Zeiss AxioCam, Germany) and the positive cells were counted using Image J.

### Western blot (WB)

Total protein was extracted with radioimmunoprecipitation assay (RIPA) lysis buffer. A volume containing 20 μg protein was loaded onto a 10% sodium dodecyl sulfate polyacrylamide gel electrophoresis (SDS-PAGE) gel, electrophoresed and transferred to polyvinylidene difluoride (PVDF) membrane. Then the membrane was blocked with 5% BSA, incubated with iNOS (BD, 1:4000), Arg1 (Santa, 1:500), Tuj-1 (Abcam, 1:1000), Olig2 (Abcam, 1:2000), GFAP (Abcam, ab 7260, 1:10000), PPARγ (Santa, sc-7196, 1:1000), or β-catenin (Abcam, ab 32572, 1:10000) primary antibody at 4°C overnight, and then incubated with secondary antibodies at room temperature for 2 h. Images were developed using a gel imaging system.

### Real-time polymerase chain reaction (RT-PCR)

The primer was designed and synthesized by Shanghai Sangon Biological Engineering Technology & Services Company. Total ribonucleic acid (RNA) was extracted using the TRIzol method, and the concentration and purity were detected using the TaKaRa quantitative PCR kit according to the reagent instructions. The reverse transcription and amplification system was performed under the reverse transcription conditions of 42°C for 15 min and 85°C for 5 sec and the amplification conditions of 95°C for 30 sec followed by 40 cycles of 95°C for 5 sec and 60°C for 30 sec; the resulting images were developed using a gel imaging system. The related primers were designed as follows:

**Table:** 

	forward primer	reverse prime
iNOS	GGCACAGGGTCATCA TCAAA	TCAGGTCACTTTGG TAGGATTT
CD86	GTAGAGTCCAGTTGTT CCTGTC	TGGTTCTGTACGAG CACTATTT
arginase-1	GTCCCTAATGACAGCT CCTTT C	CCACACTGACTCTT CCATTCTT
CD206	GTGGTCCTCCTGATTG TGATAG	CACTTGTTCCTGGA CTCAGATTA

### ELISA

Microglia under the three culture medium conditions were cultured for 24 h, then the media was replaced, and serum-free culture media was added to continue culturing. After 24 h, supernatants were collected from each group. The supernatants were collected and evaluated in duplicate using 15d-PGJ_2_ ELISA kits in accordance with the manufacturer's guidelines. After preparing the standard samples, those to be tested were then incubated for 30 min at 37°C. After repeated washings in PBS, the ELISA reagents were added, followed by a 30-min incubation at 37°C. Finally, the developing solution was added, and the absorbance of each well was measured at 450 nm in a microplate reader. The average absorbance values for each set of standards and samples were calculated, and a standard curve was constructed. The concentrations of the samples were calculated from the standard curve.

### LC-MS

Different phenotypes of microglia supernatants were acidified with HCl (1 N) to pH 3.0 and extracted with ethyl acetate. With 12 v or 15 v voltage, we divided the solvent system (315.3 molecular weight, M-H^−^) into the quantitative ion (271.2 molecular weight) and the qualitative ion (203.1 molecular weight), respectively. LC-MS/MS system (Thermo Fisher Agilent, 641013) was used to abtain the data. As an internal control to calculate extraction efficiency, 200 ng of deuterated 15d-PGJ_2_ (d4) (sigma) was used, which showed a 70% extraction efficiency. All experiments were performed by the Third Military Medical University Biomedical Analysis and Test Center.

### Data analysis

All data were presented as the mean ± standard error. SPSS16.0 software (SPSS Inc, Chicago, IL) was used for statistical analysis. One-way ANOVA with the appropriate Tukey's post hoc post hoc test was used to compare experimental groups. *P < 0.05 was considered statistically significant.

## SUPPLEMENTARY MATERIALS FIGURE


