# Supplementary material for: M2 microglia promotes neurogenesis and oligodendrogenesis from neural stem/progenitor cells via the PPARγ signaling pathway
Source: Oncotarget. 2017 Feb 28;8(12):19855–65. doi: 10.18632/oncotarget.15774 (PMC5386728; doi:10.18632/oncotarget.15774)
Supplement: Supplementary file 1 [file oncotarget-08-19855-s001.pdf]

M2 microglia promotes neurogenesis and oligodendrogenesis from neural stem/progenitor cells via the PPARγ signaling pathway

Supplementary Materials

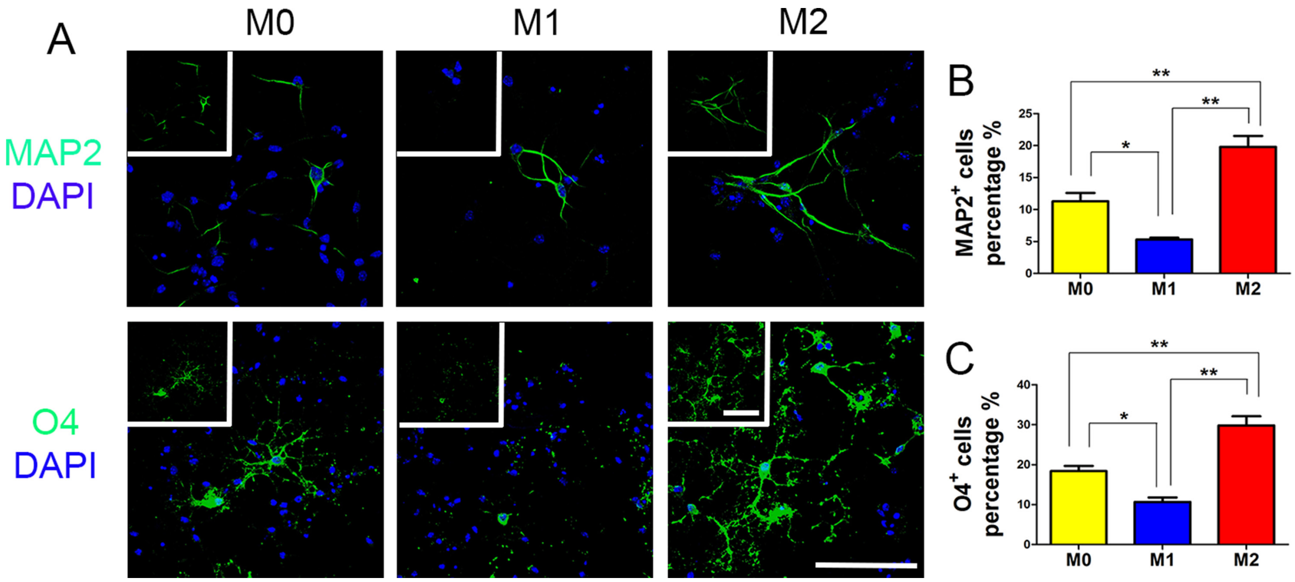

Supplementary Figure 1: NSPCs differentiation induced by different microglia phenotypes. M0, M1, and M2 supernatants were separately collected and added to 1% FBS to induce NSPCs differentiation for 21 days. (**A**) Immunofluorescence was used to evaluate the terminal differentiation markers of NSPCs. (**B**, **C**). Statistical analysis for the differentiation percentage of neurons (MAP2) and oligodendrocytes (O4) from NSPCs, respectively. $N = 8$, bar = 50 µm. $^{*}P < 0.05$, $^{**}P < 0.01$.

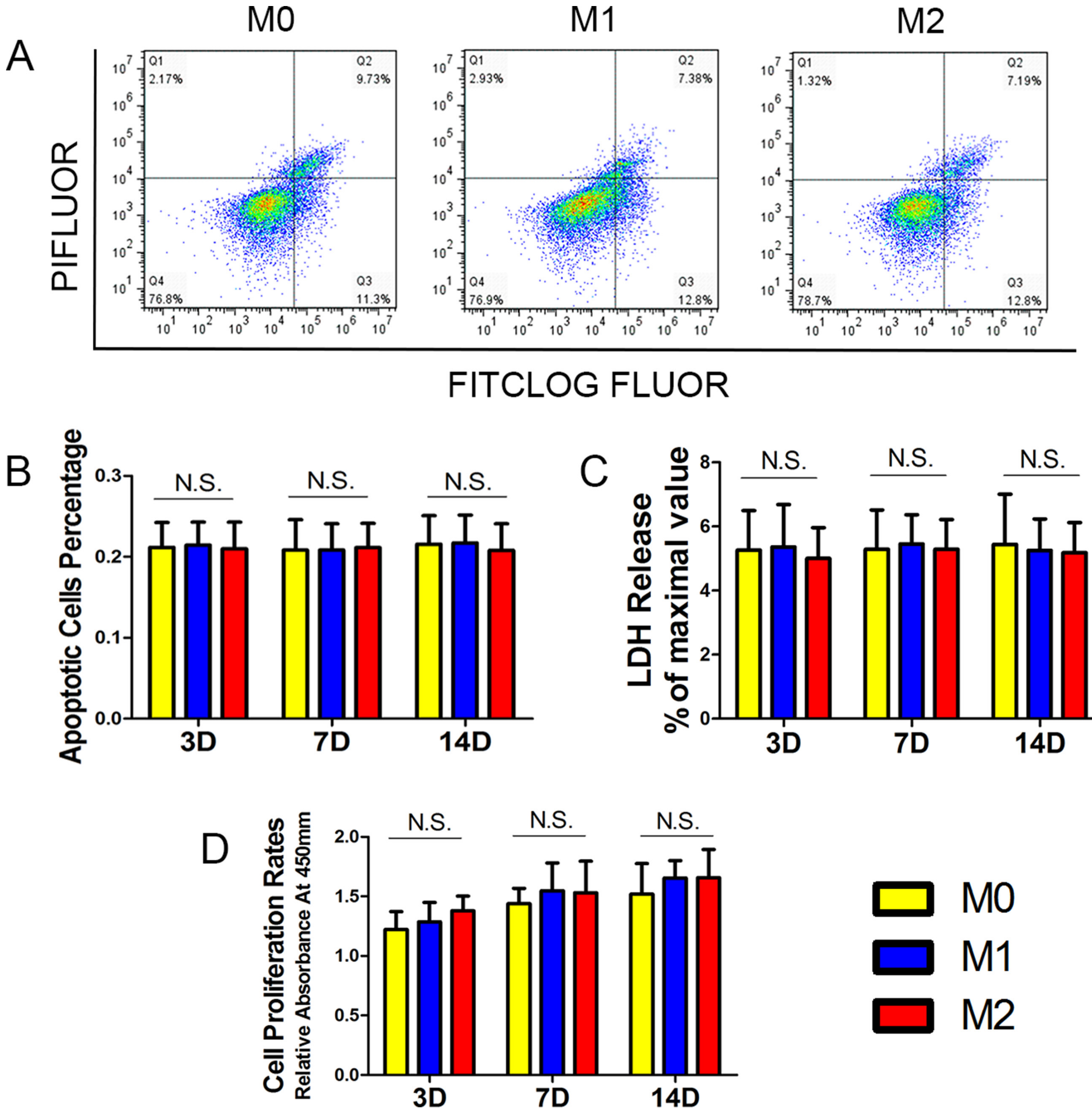

Supplementary Figure 2: NPSCs death/survival and proliferation with different phenotype microglia supernatants. (**A**) Flow cytometry showed death of NSPCs growing on M0, M1 and M2, respectively on day 14. Live cells (Q4) are both annexin V and PI negative. The cells bind annexin V while still excluding PI are at early stage of apoptosis (Q3). The cells bind annexin V-FITC and stain brightly with PI are at late stage of apoptosis (Q2). (**B**) Summarized graph showing the quantitative results from flow cytometry assay on day 3, 7 and 14. (**C**) LDH Release Assay. (**D**) Cell Proliferation Assay. $N = 8$. N.S. indicates no significant difference among M0, M1 and M2 microglia.
